# Analyzing the Effect of Zr, W, and V Isomorph Framework Substitution on ZSM-5 and Beta Zeolites for Their Use as Hydrocarbon Trap

**DOI:** 10.3390/molecules28124729

**Published:** 2023-06-13

**Authors:** Gema Gil-Muñoz, Juan Alcañiz-Monge, María José Illán-Gómez

**Affiliations:** MCMA Group, Department of Inorganic Chemistry and Materials Institute (IUMA), Faculty of Sciences, University of Alicante, Ap. 99, E-03080 Alicante, Spain; gemagilmunoz@gmail.com (G.G.-M.); illan@ua.es (M.J.I.-G.)

**Keywords:** zeolite, isomorph framework substitution, hydrocarbon traps

## Abstract

This work evaluates the effect on the adsorption and desorption kinetics of propene and toluene (used as probe molecules for vehicle cold-start emissions) of the isomorph framework substitution of Zr, W, and V on commercial ZSM-5 and beta zeolites. TG-DTA and XRD characterization data indicated that: (i) Zr does not modify the crystalline structure of the parent zeolites, (ii) W develops a new crystalline phase, and (iii) V causes the breakdown of the zeolite structure during the aging step. The CO_2_ and N_2_ adsorption data revealed that the substituted zeolites present a narrower microporosity than pristine zeolites. As a consequence of all these modifications, the modified zeolites feature different adsorption capacity and kinetics of HCs, so, different hydrocarbon trapping ability than pristine zeolites. However, a clear correlation is not observed between the changes in the porosity/acidity of zeolites and the adsorption capacity and kinetics, which depends on: (i) the zeolite (ZSM-5 or BEA), (ii) the hydrocarbon (toluene or propene), and (iii) the cation to be inserted (Zr, W, or V).

## 1. Introduction

The accomplishment of current and future legislation requires a huge increase in the average fuel economy and that poses an unprecedented challenge for the automotive industry. Thus, new-generation internal combustion engines, especially gasoline vehicles that are less energy efficient than diesel engines, will have to be much more efficient. The increase in efficiency means that more energy must be extracted from the fuel, which necessarily implies a significant reduction in the exhaust gas temperature [1]. Due to this decrease in exhaust gas temperature, the current three-way catalyst formulations (TWCs) will not meet the strict emission limits imposed by current or future legislation, as they require a temperature between 200 and 300 °C to achieve their optimal levels of gaseous pollutant conversion. Furthermore, the problem is aggravated during cold-start periods as, due to the low temperature of the exhaust gases, a longer time is needed to reach the temperature for the optimum operation of the TWC [2]. Thus, during the cold-start regimen around 80% of the exhaust hydrocarbon (HCs) is evolved [3]. In this regard, it has been proposed to use a storage system upstream of the TWC (called storage traps or HCs traps) for retaining the harmful gases until the optimum operating temperature of the TWC is reached.

The technology developed for the control of cold-start gases emitted during the regime is based on the use of an adsorption system able to capture the pollutants emitted at low temperatures until the TWC reaches its optimum operating temperature, at which the species will be desorbed and catalytically removed [2]. Several adsorption systems are being proposed, showing zeolites to have the best performance since, due to their high specific surface area and tunable size of microporosity, they are suitable for the adsorption of small molecules [2]. The studies about HC adsorption on zeolite concluded that both pore texture and chemical composition determine the HCs’ trap performance. Among the zeolites analyzed, ZSM-5 and beta zeolites are one of the most suitable for this application [4,5]. However, these zeolites present, as a main drawback, a limited service life due to the humidity and temperature conditions of the exhaust gases. So, the actual challenges are the increase in hydrothermal stability of zeolites and of the HCs’ adsorption capacity by modifying their chemical composition [2,6,7].

In order to determine the performance of zeolites as HCs trap, simulated cold-start adsorption and temperature-programmed desorption (TPDs) experiments, using propene and toluene as light and heavy HC probe molecules for hydrocarbon evolved by gasoline automobile engine, are usually performed [8,9]. As for light HCs, the adsorption capacity and desorption temperatures do not reach the target values due to the lower adsorption strength in the porosity of zeolites, and different strategies have been proposed to increase the adsorption strength. Thus, in many studies, ion-exchanged zeolites (using different cations such as Li, Na, Cs, Cr, Cu, and Ag [10,11,12]) are employed. Other studies propose the use of transition metals, such as Cu, Ag, and Pt, as they improve the adsorption capacity of both unsaturated light HCs and aromatic HCs (i.e., toluene), by the interaction of the metallic species with the π-electrons of the unsaturated HCs [11,13]. Another strategy to modify the chemical composition of zeolites is the isomorphous framework substitution, consisting of the introduction of transition metal cations in the regular tetrahedral framework sites. This approach has been extensively used for the development of numerous catalyst-based zeolites [14,15,16,17]. The substitution can be achieved during hydrothermal synthesis [14] or by ion-exchanged [15] in a post-synthesis step [16,17]. In most cases, the transition metal substituted zeolites obtained by direct synthesis show a lower pore texture development, whereas those obtained by post-synthesis method hold a pore texture such as raw zeolites. Hence, for using zeolites as HC traps, the post-synthesis substitution method seems to be more convenient.

Considering the above background, this work aims to analyze the use of Zr, W, and V for isomorphous framework substitution of ZSM-5 and Beta commercial zeolites that will be used as HC traps. Zr has been selected because it is one of the metals broadly used for the isomorph framework substitution of zeolites, because it allows higher values of heteroatom incorporation [14,16,17]. In contrast, W and V (that have been scarcely analyzed for adsorption applications) will be used because: (i) they have high affinity toward unsaturated HCs shown by V and, (ii) due to the improvement of the hydrophobicity of zeolites featured by W.

## 2. Results and Discussion

### 2.1. Preparation and Preliminary Characterization of Samples

In Table S1 of the Supplementary Material, the elemental analysis and the acidity of zeolites are shown. In agreement with other studies [16,17], the Si/M ratio (being M Al, Zr, W, or V) corresponds with that of pristine zeolites, indicating a total substitution of Al by M cations on the zeolite framework. Infrared spectra of all samples (Figure S1 Supplementary Material) are almost identical to those obtained for pristine zeolites, suggesting that M cations are localized on the Al^+3^ sites. The acidity data reveal that the dealumination-substitution steps generate a slight reduction in the amount of acid sites for Zr and V cations, whereas, for W, a higher decrease is detected (i.e., from 1820 to 340 μmol NH_3_/g, from B to BW). Finally, the aging treatment causes a large decrease in acid sites on samples.

**Figure 1:**
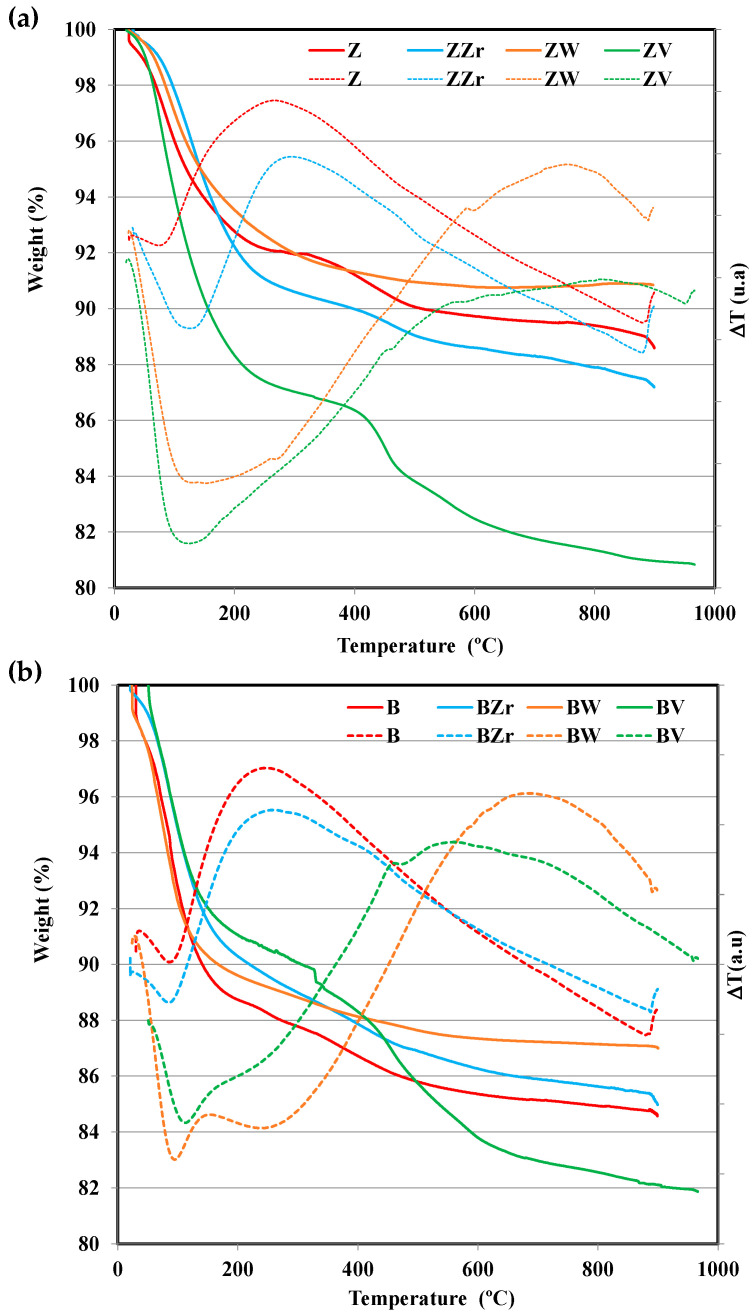
Thermogravimetric (continuous line) and differential thermal analysis (dotted lines) profiles for (**a**) fresh, Zr-, W-, and V-substituted Z zeolites, (**b**) fresh and Zr-, W-, and V-substituted B zeolites.

The dealuminated zeolites and the Zr-, W-, and V-substituted zeolites were analyzed by the TG-DTA technique, and, Figure 1 shows the thermogravimetric (TG) and differential thermal analysis (DTA) profiles for all samples. 

All samples show a similar initial (up to ca. 200 °C) mass loss, corresponding to the desorption of the physisorbed water, and, most likely, to the physisorbed atmospheric CO_2_ [18]. The second stage (up to ca. 500 °C) corresponds to the release of some structural water molecules corresponding to the loss of terminal hydroxyl groups, which could be related to the incorporation of the M cations on the zeolite lattice [18,19]. Interestingly, whereas ZZr and BZr zeolites follow similar TG and DTA profiles, for raw zeolites (ZSM-5 and BEA), W-zeolites, and V-zeolites, different profiles are observed. Thus, at temperatures higher than 500 °C, W-zeolites show the lowest weight loss, and even feature a small weight increase for ZW. In contrast, Zr-zeolites show a continuous weight loss until 900 °C, whereas V-zeolites show a higher weight loss. The elemental analysis data for V-zeolites (see Table S1 Supplementary Material) reveal a higher reduction in the V content for aged samples. The V and W release from vanadium-based SCR catalysts has already been reported [20,21], which was related to the formation of vanadium or tungsten oxides. The small weight loss shown by W-zeolites reveals the high thermal stability of W in both zeolites. DTA curves also show a clear difference between Zr-zeolites and W-zeolites and V-zeolites. Thus, DTA curves of W-zeolites and V-zeolites show an endothermic band until 500 °C, whereas the pristine zeolites and Zr-zeolites show an exothermic band. Note that the exothermic band located around 500–800 °C, which could be related to the structural change on the zeolite framework, is most clear for W-zeolites.

**Figure 2:**
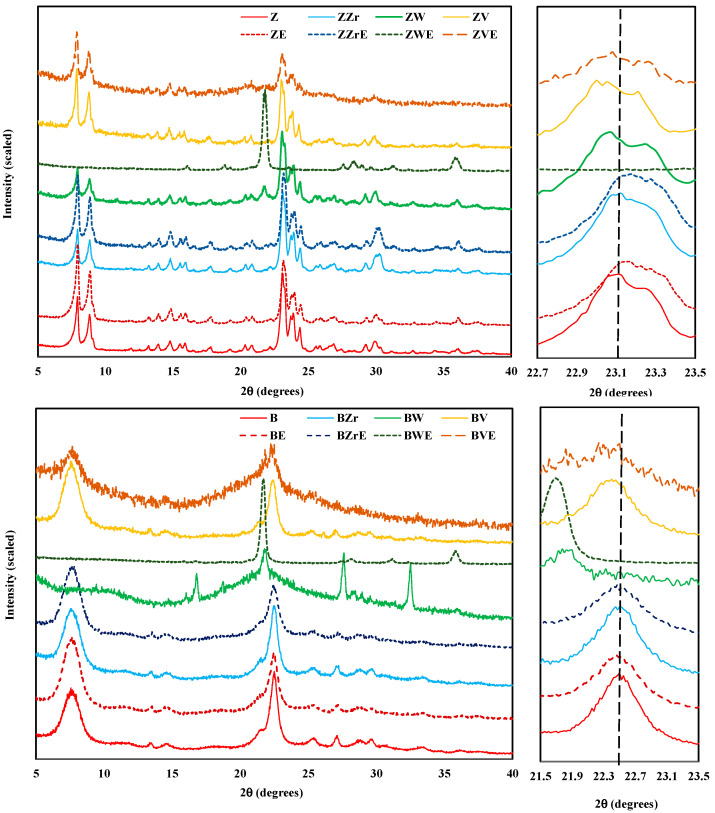
Powder X-ray diffraction patterns of fresh and aged H-form and Zr-, W-, and V-modified zeolites.

To obtain information about the structural changes, all zeolites (H-form zeolites and the Zr-, W-, and V-substituted, both fresh and aged) were analyzed using powder X-ray diffraction; the XRD patterns are shown in Figure 2. The XRD patterns of almost fresh samples show well-defined peaks indicating that the original crystal structure has been retained. However, BW zeolite appears to be an amorphous material as a broad band in the range of 2θ = 15–30° is detected. Additionally, this sample shows four new prominent peaks (regarding pristine B zeolite), suggesting the development of a new crystalline phase amongst the amorphous phase of the original zeolite framework. In the case of ZW, a small new peak around 21° is also observed. Zr-modified (fresh and aged) zeolites show XRD patterns identical to the Z and B parent zeolites. Thus, these results indicate that, in agreement with other studies [17,22,23,24], the incorporation of zirconium into the framework of zeolites does not modify the crystalline structure of the parent zeolites. On the contrary, W and V incorporation modify the framework of zeolites: (i) W causes the development of a new crystalline phase, with well-defined crystalline peaks on the aged sample and, (ii) for V, a breakdown of the zeolite structure takes place during the aging step (forming an amorphous solid) which could be due to the V released as it is revealed by the significant weight loss (see Figure 1) and by elemental analysis (see Table S1). 

On the right of Figure 2, the magnification of the main reflection peaks of fresh ZSM-5 051/501 [25] and BEA 302 [26], modified and aged zeolites are shown. The aging step originated a smooth contraction of the framework of ZSM-5 samples, as the peak position is shifted to higher 2θ, but, for BEA samples the peaks do not change. On the other hand, zirconium incorporation into the framework changes the peak position of both zeolites, which reveals the successful substitution of Al by Zr [24]. In contrast, W and V incorporation provoke a slight peak shift toward lower 2θ values, suggesting an elongation of the framework structure. Hence, the loss of crystallinity for BW, BE, and ZVE samples and the formation of a new crystalline phase for ZWE and BWE reveal the effect of W and V substitution, as well as of the aging step, on the zeolite framework. Note that the DTA exothermic band registered (Figure 1) for W- and V-modified samples, supports the idea of a recrystallization process.

**Figure 3:**
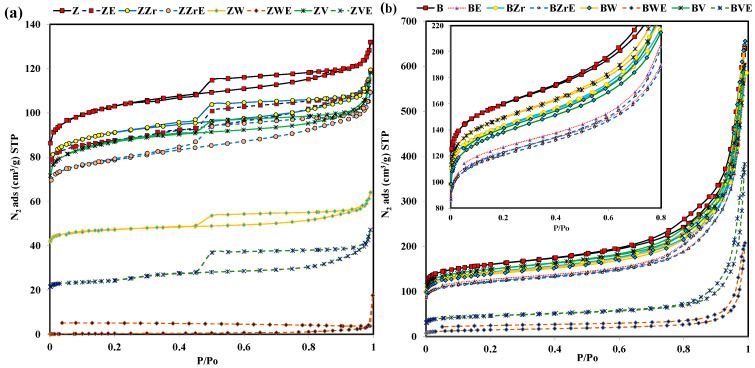
N_2_ adsorption–desorption isotherms at −196 °C of fresh and aged (**a**) ZSM-5 series, (**b**) BEA series.

The analysis of the pore texture of fresh and aged zeolites was carried out by N_2_ adsorption at −196 °C and CO_2_ adsorption at 0 °C. Figure 3 shows the N_2_ adsorption–desorption isotherms obtained at –196 °C for the series of zeolites and Table 1 lists the textural properties. The most relevant points to analyze in these isotherms are: (i) the magnitude of adsorption at P/P_o_ < 0.3, which is related to the specific micropore volume, (ii) the sharpness of the isotherm knee, related to the micropore size distribution, (iii) the slope at P/P_o_ > 0.3, which informs about mesoporosity, and (iv) the presence of a hysteresis loop, which is due to the presence of mesopores. Thus, it is observed that all zeolites, except aged W-samples, present a significant adsorption capacity at low P/P_o_ values, indicating the presence of micropores [27]. In addition, BEA samples show well-developed mesoporosity [27], as deduced not only from the sharp step of the isotherm in the 0.75–0.95 P/P_o_ region but also from the presence of the hysteresis loop, which seems to be an H3 type due to the presence of aggregated platy particles [27]. For ZSM-5 samples, the N_2_ adsorption isotherm also shows an H2 type hysteresis loop which reveals the presence of an interconnected network of pores having a relatively low adsorption capacity in the 0.3–0.96 P/P_o_ region, as the specific mesopore volume is relatively low.

Regarding the effects of post-treatments, both series of zeolites show a similar trend. Thus, the dealumination and substitution treatments generate a reduction in the N_2_ adsorption capacity in the 0–0.2 P/P_o_ region (Figure 3), mainly related to the microporosity. Consequently, all samples show a decrease in the BET-specific surface area (see Table 1). For the BEA series, presenting a considerable mesoporosity, the specific mesopore volume also decreases (between 7 and 2%), being the degree for WB higher than for WB. Note that the aging process causes a high reduction in the N_2_ adsorption capacity in both zeolite series due to a high reduction in the micropore volume, mainly for W and V-zeolites (see Table 1). Puértolas et al. [7] reported a similar micropore reduction on aged ZSM-5 and BEA zeolites. These authors, in agreement with Riberio-Carrot et al. [28], concluded that the aluminum released from the framework during aging causes the observed micropore reduction. In this work, as all of the zeolites have been dealuminated before the aging treatment, the micropore decrease must be related to structural changes. This fact is more relevant for W, as the aging step provokes the formation of a new crystalline phase (Figure 2), which results in the loss of porosity found. For V, the release of volatile oxide seems to generate the collapse of micropores. Finally, according to other authors [24], for Zr samples, the post-treatment does not significantly modify the porosity of pristine zeolites. 

Further information about the effects of substitution on the size of the pores has been obtained from a detailed analysis of the specific pore volumes shown in Table 1. It is important to keep in mind that the specific micropore volume calculated from N_2_ adsorption data (V_N2_) informs about the whole range of micropores (pore size up to 2 nm), while the CO_2_ adsorption data (V_CO2_) only inform about the narrowest micropores (pore size < 0.7 nm) [29]. For the ZSM-5 series, as the V_CO2_ is higher than the V_N2_, a narrow microporosity is present. However, for BEA series, both volumes are similar and they decrease after the dealumination, substitution, and aging treatments. Nevertheless, whereas in the BEA series, the micropore volume reduction is similar, for ZSM-5 series, the dealumination and substitution scarcely reduce the V_CO2_ (except for W samples), and, after the aged step, a higher reduction in V_CO2_ than in V_N2_ is detected. In summary, the analysis of CO_2_ and N_2_ adsorption data indicated that the treatments favor the development of the narrowest microporosity concerning pristine zeolites, being higher on ZSM-5 samples.

### 2.2. HC Adsorption–Desorption Tests

#### 2.2.1. Single Toluene and Propene Adsorption–Desorption Tests

**Figure 4:**
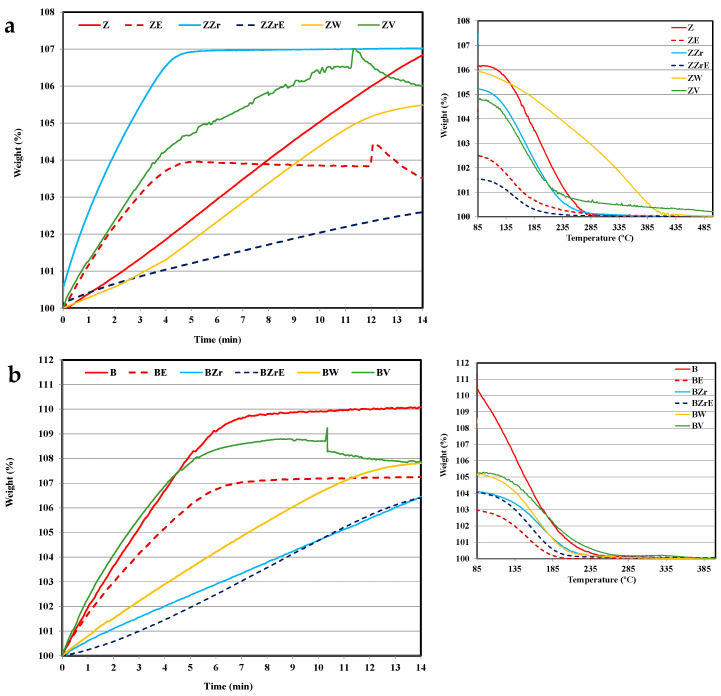
Toluene adsorption–desorption isotherms at 80 °C. (**a**) ZSM-5 series and (**b**) BEA series.

**Figure 5:**
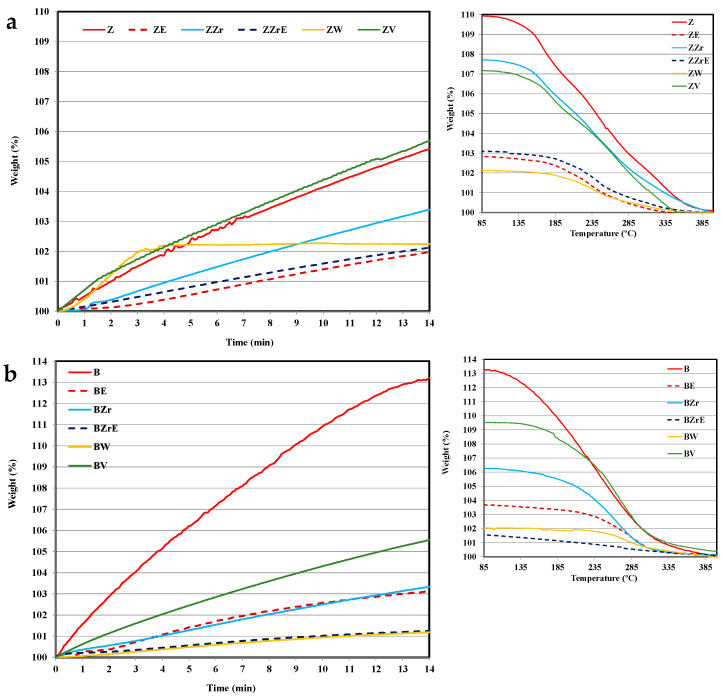
Propene adsorption–desorption isotherms at 80 °C. (**a**) ZSM-5 series and (**b**) BEA series.

Figure 4 and Figure 5 show the adsorption–desorption isotherms at 80 °C on the two series of zeolites (modified and aging samples) for toluene and propene, respectively. Note that the figures only show the data corresponding to the first 14 min of adsorption, although the tests have been extended until maximum uptake was achieved. To analyze the desorption, the samples were flushed in He followed by heating from 80 to 500 °C, and Figure 4 and Figure 5 show the temperature-programmed desorption profiles (TPD).

Before starting the discussion, it should be considered that concerning the cold-start application, the most relevant information is how an adsorbent material’s performance corresponds during the first two minutes [2,30]. Thus, the adsorption profiles at longer times only provide information about how far the equilibrium is. First of all, it is clear that the substitution and aging treatments provoke a clear modification in their adsorption capacity and kinetics of pristine zeolites and, that the changes seem to depend on: (i) the zeolite, (ii) the hydrocarbon, and (iii) the cation inserted. 

Thus, focusing our attention during the first two minutes of adsorption, it seems that BEA zeolites present a better performance than ZSM-5 zeolites, and the propene adsorption kinetic is slower than toluene. Concerning the effect of metal used for substitution, ZrB zeolite shows the lowest adsorption rate for both hydrocarbons, but, the porosity and acidity of Zr-modified zeolites are similar to that of pristine zeolites (Table 1 and Table S1). The incorporation of W into ZSM-5 allows the highest adsorption rate of propene, despite its very low acidity concerning pristine zeolite (i.e., 392 vs. 2100 μmol NH_3_/g, ZW vs. ZSM-5), whereas W-BEA shows the lowest adsorption rate, but the highest modification of porosity with respect to BEA zeolite (Table 1). Finally, note that the incorporation of V improves the performance for both HCs and both ZSM-5 and BEA-modified zeolite samples.

On the other hand, the aging treatment affects propene adsorption kinetic more than toluene adsorption kinetic, and for ZSM-5 an increase in the adsorption kinetics is observed. However, W and V aged zeolites almost do not adsorb (and they are not included in the figures), in agreement with their very low porosity (see Table 1). 

Regarding the desorption process, it seems that it is not significantly affected by substitution or aging treatments as the profiles are quite similar (see Figure 4 and Figure 5), showing propene with a slower adsorption kinetic and a higher desorption temperature than toluene. Similar results have been reported by Burke et al. for BEA zeolites [31]. Note that, for the cold-start application, the desorption could take place at higher temperatures. Considering this fact, ZW and ZV seem to be interesting for cold-start application for toluene since the desorption occurs over a wider temperature range. For W, the high temperature for toluene desorption could be related to its small porosity size (see Table 1), which could also justify the slow adsorption kinetics (Figure 4). However, VZ shows fast toluene adsorption and desorption kinetic, which is related to the fraction of toluene strongly adsorbed, most probably, on the V cation sites of the zeolite framework. In this regard, it must be pointed out that only ZV and BV zeolites change to black color after the desorption test due to the coke deposition generated by the thermal recombination, oxidation, or the cleavage of toluene chemisorbed on V sites. The coke deposition has been confirmed by the presence of a characteristic graphitic G band on Raman spectra (see Figure S2 Supplementary Material). The deposition of coke has been observed by other authors on beta and ZSM-5 zeolites doped with Co, Mn, or Fe [32,33]. Kobatake et al. [34] called these zeolites “Super hydrocarbon Reformer Trap” as they present the dual role as storage HCs and as catalysts to their removal. Contrarily, Jang et al. [35] reported the HCs’ oxidation with Cu-impregnated BEA zeolites.

#### 2.2.2. Multicomponent Toluene and Propene Adsorption–Desorption

**Figure 6:**
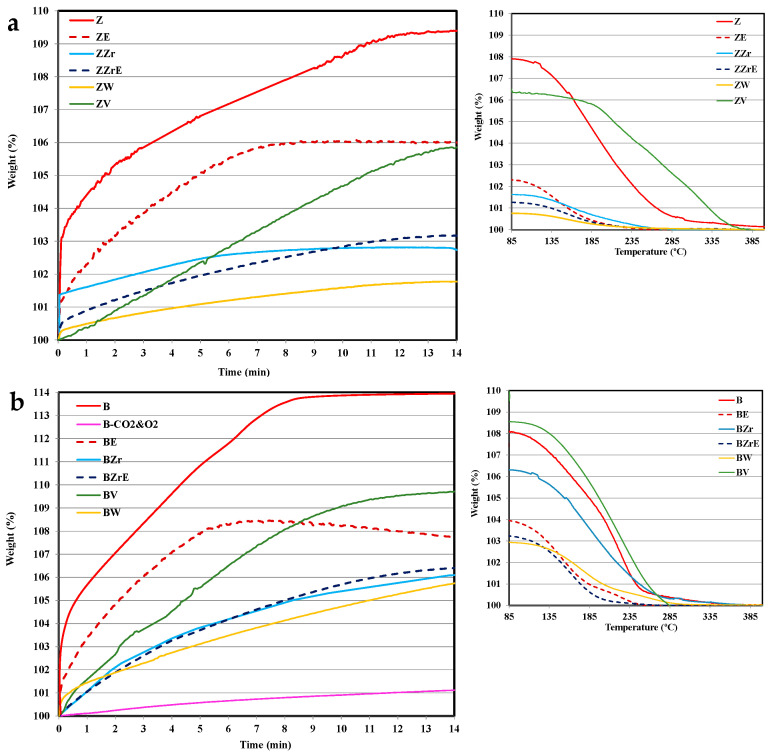
Multicomponent adsorption–desorption at 80 °C on zeolites. (**a**) ZSM-5 series; (**b**) BEA series.

To deeply analyze the performance of zeolites for the HCs captured from vehicle exhaust, the next step is to study the HC uptake using a gas flow containing a mixture of the two HC models (propene and toluene). Figure 6 shows the adsorption and desorption profiles of pristine and modified zeolites at 80 °C under a flow composed of toluene, propene, CO_2_, and O_2_. 

It is well known that in a multi-component gaseous mixture, the adsorption of each molecular species will take place in the same micropore volume. Consequently, the adsorption capacity achieved for each HC in the multi-component test must be lower than the addition of the adsorption capacity determined for each HC during a single test (see Figure 4, Figure 5 and Figure 6). Thus, as an example, ZZr shows an uptake on the single toluene and propene corresponding to an increase in weight of 8 wt. % and 6 wt. %, respectively, whereas during the multicomponent test, it is 3 wt. %. This low value could be related to the presence of CO_2_ as the main component of the gas mixture (12% vol, vs. 0.08% vol. and 0.15% vol of toluene and propene, respectively), so the adsorption of CO_2_ will limit the adsorption of hydrocarbons. To probe this hypothesis, an additional test was carried out using a mixture composed of CO_2_ and O_2_ (in the same% vol as that used in the multi-component test) and, it is concluded that the adsorption of CO_2_ is relatively low as only an increase of 1% in weight is detected. Thus, the competitive adsorption between toluene and propene causes a decrease in the adsorption capacity in the multi-component gas mixture. However, in the case of Ag-ZSM5, Lee et al. [36] reported minor competitive adsorption. In line with this, Azambre et al. related the adsorption decrease to kinetic limitations due to mutual steric hindrance during the hydrocarbon diffusion within the internal porosity [37]. 

Additionally, it is observed that the adsorption kinetics seem to also be affected by the coexistence of the two hydrocarbons in the same gaseous mixture. Thus, pristine and Zr-based zeolites show higher adsorption kinetics than that observed for single adsorption, whereas V- and W-based zeolites show an adsorption kinetic between those detected for single toluene and propene adsorption. Regarding desorption, for most of the zeolites the desorption process requires an intermediate temperature than that observed for single adsorption (see Figure 4 and Figure 5). Westerman and Azambre reported a similar trend in the desorption temperature of propene and toluene on single and mixed gas flows [38]. 

**Figure 7:**
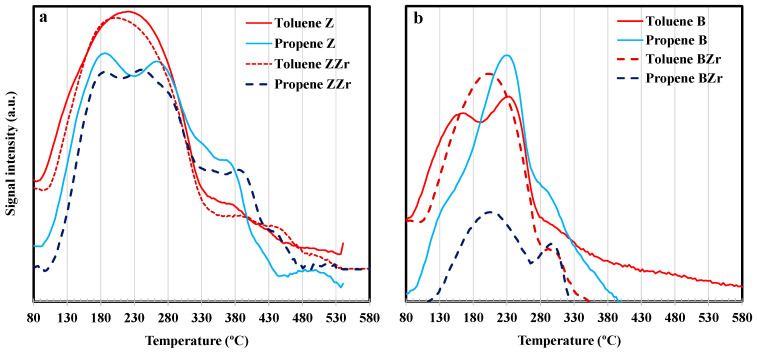
Toluene and propene thermodesorption profiles on (**a**) ZSM-5 and Zr-ZSM-5; (**b**) BEA and Zr-BEA.

Finally, the total amount of propene and toluene adsorbed was obtained from the amount of hydrocarbon desorbed during the TPD experiments analyzed by mass spectrometry by following the peaks at m/e 41 for propene and m/e 91 for toluene. As an example, Figure 7 shows the desorption profiles on pristine Zr-modified zeolites. 

By comparing the ratio corresponding to the increase in weight for toluene/propene shown by Z and B zeolites at the beginning of DTP after a single adsorption test (106/110 and 110/113 for Z and B, respectively, see Figure 5 and Figure 6) and the ratio between the area of toluene and propene desorption shown in Figure 7, it can be stated that the desorption of toluene and propene adsorbed from a multicomponent mixture is similar to that observed after single adsorption. However, as can be observed in Figure 7, the Zr substitution causes the preferential adsorption of a particular hydrocarbon, which is different depending on the zeolite. Thus, using the same comparison previously employed (i.e., between toluene/propene weight ratios on a single adsorption test and those shown in the multi-component test) it seems that BZr preferentially adsorbed toluene. This different behavior between B and BZr is an intriguing subject since both show similar pore textures (Table 1) and acidity (Table S1). 

## 3. Materials and Methods

### 3.1. Zeolites and Chemicals

Commercial ZSM-5 (Si/Al 23) and Beta (BEA) (Si/Al 25) zeolites were provided by Alfa Aesar (Thermo Fisher Scientific) in the ammonium form and, to obtain the protonated form, they were calcined at 550 °C in the air for 2 h. Both zeolites have a similar Si/Al ratio, so, the effect of different content on Al could be discarded in the zeolites during the dealumination process. The protonated forms were used as raw material for post-synthesis treatments and, as cationic precursors Zr(NO_3_)_4_·5H_2_O (Sigma-Aldrich), WO_3_·H_2_O (Wako Pure Chemical Industries, Ltd.), and VOSO_4_·5H_2_O (Sigma-Aldrich) were employed.

### 3.2. Zeolites Treatments

The modification of zeolites was carried out following the two-step process reported elsewhere [14,39,40]: (i) an acid dealumination of the commercial zeolite, to create vacant T-atom sites and (ii) a cation incorporation via solid-state exchange or liquid-phase substitution. Thus, protonated BEA and ZSM-5 were dealuminated by a standard literature procedure (20 mL of concentrated HNO_3_ per gram of zeolite, 80 °C, and 12 h) [20,39,40,41]. The resulting materials were centrifugated, washed with deionized water, and dried at 150 °C overnight, obtaining the dealuminate form of zeolite (denoted as deAl-zeolite). 

For the incorporation of Zr, W, and V cations, the deAl-zeolite (1.0 g) was suspended in an ethanol solution (100 mL) with 0.7 mmol of cationic precursor (Zr(NO_3_)_4_·5H_2_O (0.294 g); WO_3_·H_2_O (0.171 g); VOSO_4_·5H_2_O (0.175 g)). This slurry was refluxed for 8 h and, then, it was heated under stirring until dry, washed with ethanol, and dried at 100 °C. Finally, the powder was calcined (1 °C min^−1^, 550 °C, and 6 h) [22,23,41,42]. The nomenclature includes the initial zeolite (X-deAl-zeolite, being Z or B for ZSM-5 and BEA, respectively) following the cation incorporation (Zr, W, and V).

All samples were hydrothermally aged by heating to 900 °C in a 12% CO_2_/10% H_2_O/1% O_2_/77% N_2_ mixture for 4 h in a fixed bed reactor. These conditions are roughly equivalent to 200,000 km of driving. The nomenclature of aged samples includes the letter “E” at the end of the sample name.

### 3.3. Zeolites Characterization

Elemental analysis of the zeolite was performed by inductively coupled plasma atomic emission spectroscopy (ICP-AES) of a solution of zeolites in HF (see Table S1 Supplementary Material). 

Zeolite acidity was measured by ammonia chemisorption using a Micromeritics ASAP2705 provided with a TCD. For the experiment, 80 mg of zeolite was exposed to an ammonia flow of 40 mL/min (5% NH_3_/He) at 120 °C for 30 min. After that, the gas ammonia flow was changed to a He flow (40 mL/min) to remove the weakly adsorbed ammonia and then the sample was heated at 10 °C/min up to 550 °C. Before the chemisorption analysis, zeolites were pretreated by heating at 550 °C for 30 min in synthetic air (see Table S1 Supplementary Material).

Infrared spectroscopy (diffuse reflectance infrared Fourier transform spectroscopy—DRIFTS) was used to study the zeolite. The spectra were continuously recorded (Jasco FTIR 4700 IRT 5200) at a resolution of 4 cm^−1^, in the interval of 4000–400 cm^−1^, with 60 scans averaged.

To follow the performance of the dealuminate and substituted zeolites during the calcination step, thermogravimetric and differential thermal analysis (TG-DTA) experiments were carried out using a thermobalance from TA Instruments (SDT 2960). To develop the experiments, 10 mg of sample was heat-treated (20 °C/min up to 900 °C) in a synthetic air (60 mL/min). 

The crystal structure of zeolites was examined by powder X-ray diffraction (XRD) analyses using a Seifert JSO Debye-Flex 2002 diffractometer equipped with Cu Kα radiation. Powdered diffraction patterns were recorded between 5 and 40° (2θ) using increments of 0.025° and a counting time of 3 s.

The analysis of the porous texture was carried out by N_2_ adsorption at −196 °C and CO_2_ adsorption at 0 °C. Adsorption isotherms were carried out in an Autosorb 6 equipment from Quantachrome. Before the adsorption test, the samples were degassed at 250 °C under a vacuum (1 Pa) for 4 h. The distribution of specific pore volumes was calculated as follows [29]: (i) the volume of narrow micropores (pore size < 0.7 nm) by applying the Dubinin–Radushkevich (DR) equation [43] to the CO_2_ adsorption data at relative pressures < 0.015; (ii) the total micropore volume (pore size < 2 nm), which includes the volume of the narrow micropores and supermicropores, by applying the DR equation to the N_2_ adsorption data at relative pressures < 0.14; and (iii) the volume of mesopores, by applying the BJH method to the N_2_ adsorption data [44]. The specific surface area was determined using the BET [45] (Brunauer–Emmett–Teller) equation.

The D bands of the ZV before toluene DTP experiments were also analyzed using Raman spectrometry (dispersive Horiba Jobin-Yvon: LabRAM with a 600 grooves/mm grating, DPSS 325 nm laser and 1 mW of power on the sample, and a confocal microscope with a 10× objective and 0.25 numerical aperture).

### 3.4. HCs Adsorption and TPD Tests

The trapping performance and desorption kinetics of the zeolites were analyzed using a thermobalance (TA Instruments, SDT Q600) coupled to a mass spectrometer (Balzers QMS 100). Toluene and propene were employed as probe molecules for heavy and light hydrocarbons evolved by gasoline engine automobiles. Two tests have been developed using similar HC concentrations: (i) adsorption of a single HC (propene or toluene) and (ii) adsorption of a mixture of both HCs, i.e., propene and toluene. The concentration used simulates that usually found in gasoline engine exhausts: 1500 ppm of propene and 800 ppm of toluene. In the multicomponent experiments, the gas mixture includes 12% vol CO_2_ and 1% vol O_2_ in addition to HCs. The propene concentration was adjusted from a >99.5% propene (Alphagaz, Air Liquide) using a gas mixing device. Toluene (Sigma-Aldrich-34866M MSDS, 99.9%) vapors were generated by a saturator placed into a thermostated bath at 5 °C to generate the appropriate partial pressures (800 ppm) after dilution in a He low or He/CO_2_/O_2_/propene flow.

Before experiments, all samples were pretreated in situ under synthetic air (60 mL/min) at 550 °C for 30 min. After, the samples were cooled down under He to 80 °C, and HC adsorption was started (60 mL/min) until saturation was reached (for around 30 min). After saturation, the gas flow was changed to He (60 mL/min) to remove most of the weakly adsorbed HCs, and then, the temperature was increased from 80 to 600 °C at 20 °C/min under He. The gases emitted during this step were analyzed by mass spectrometry, following the mass spectral peaks at m/e 41 for propene and m/e 91 for toluene.

## 4. Conclusions

The isomorph substitution with Zr, W, and V generates different acidity, structure, and porous texture changes in ZSM-5 and BEA zeolites:

(i) Dealumination followed by the substitution provokes a low decrease in the amount of acid sites for Zr and V, and, for W, a high decrease takes place.
(ii) Dealumination and Zr and V substitution does not modify the crystalline structure of pristine zeolites, neither the porosity. However, after W substitution, a new crystalline phase and a high micropore volume reduction are detected.
(iii) The aging treatment causes a high reduction in the acidity and pore texture, mainly for W- and V-modified samples. For V samples a breakdown of the zeolite structure occurs, which could be due to the release of V, yielding an amorphous solid.

A clear correlation between the modification of zeolite properties and adsorption capacity and kinetics for single toluene or propene adsorption–desorption is not observed. These two parameters seem to depend on:

(i) the zeolite, presenting better performance for BEA-based zeolites than those based on ZSM-5.
(ii) the hydrocarbon, showing the adsorption of propene at a slower rate than that of toluene.
(iii) the cation (Zr, W, or V). The Zr incorporation into ZSM-5 allows the highest adsorption rate for toluene. The incorporation of V improves the adsorption performance for both hydrocarbons for ZSM-5 and on BEA.

Thus, ZSM-5-based zeolites substituted with W and V seem to be interesting for toluene since desorption is extended over a wide temperature range. V presents a dual role as hydrocarbon storage and as a catalyst for their removal, since the toluene chemisorbed on V sites suffers a thermal recombination and oxidation or a cleavage after desorption.

Finally, the adsorption capacity achieved during the multi-component mixture tests is lower than the sum of individual adsorption capacities, confirming competitive adsorption between toluene and propene. However, Zr samples present preferential adsorption of toluene or propene, depending on the pristine zeolite. Thus, as an example, BZr shows preferential adsorption of toluene over propene.

## Figures and Tables

**Table 1:** Textural properties of zeolites.

Zeolite	S_BET_ (m^2^/g)	V_N2_ ^1^	V_CO2_ ^2^	V_mesopore_	V_total_ ^3^
		(cm^3^/g)			
Z	380	0.16	0.22	0.03	0.20
ZE	324	0.14	0.15	0.03	0.18
ZZr	343	0.14	0.22	0.02	0.18
ZZrE	290	0.12	0.19	0.03	0.17
ZW	170	0.07	0.07	0.01	0.10
ZWE	2	0.00	0.02	0.00	0.03
ZV	330	0.14	0.21	0.02	0.18
ZVE	97	0.04	0.05	0.02	0.07
B	590	0.26	0.25	0.46	0.99
BE	450	0.20	0.18	0.43	0.90
BZr	530	0.23	0.24	0.43	0.91
BZrE	450	0.20	0.19	0.44	0.93
BW	508	0.22	0.20	0.45	1.01
BWE	3	0.01	0.01	0.11	0.32
BV	560	0.24	0.26	0.42	0.93
BVE	172	0.08	0.09	0.24	0.59

^1^ Specific total micropore volume; ^2^ specific narrow micropore volume (<0.7 nm); ^3^ specific total pore volume.

## Supplementary Materials

The following supporting information can be downloaded at: https://www.mdpi.com/article/10.3390/molecules28124729/s1, Table S1: Si/M ratio and acidity of zeolites. Figure S1. DRIFTS spectrum of Zeolites sample series of ZSM-5 and BETA. Figure S2. Raman spectra visible at 325 nm of zeolite ZV and ZV before toluene DTP. Figure S3. Kinetic curves obtained by application of analytical equation Fick’s Law (a) and the Elovich equation (b) to toluene adsorption data on ZSM-5 series. Figure S4. Kinetic curves obtained by application of analytical equation Fick’s Law (a) and the Elovich equation (b) to propene adsorption data on ZSM-5 series. Figure S5. Kinetic curves obtained by application of analytical equation Fick’s Law (a) and the Elovich equation (b) to toluene adsorption data on BETA series. Figure S6. Kinetic curves obtained by application of analytical equation Fick’s Law (a) and the Elovich equation (b) to propene adsorption data on BETA series.
