# Supplementary material for: Analyzing the Effect of Zr, W, and V Isomorph Framework Substitution on ZSM-5 and Beta Zeolites for Their Use as Hydrocarbon Trap
Source: Molecules. 2023 Jun 13;28(12):4729. doi: 10.3390/molecules28124729 (PMC10302204; doi:10.3390/molecules28124729)
Supplement: Supplementary file 1 [file molecules-28-04729-s001.zip › Suplementary Material J Alcaniz et al.pdf]

Analyzing the Effect of Zr, W, and V Isomorph Framework Substitution on ZSM-5 and Beta Zeolites for Their Use as Hydrocarbon Trap

Gema Gil-Muñoz, Juan Alcañiz-Monge * and María José Illán-Gómez

MCMA Group, Department of Inorganic Chemistry and Materials Institute (IUMA), Faculty of Sciences, University of Alicante, Ap. 99, E-03080 Alicante, Spain; gemagilmunoz@gmail.com (G.G.-M.); illan@ua.es (M.J.I.-G.)

* Correspondence: jalcaniz@ua.es

Table S1. Si/M ratio and acidity of zeolites

Zeolite	Si/M [a] (mol/mol)	Acidity [b] (μmol/g)
**Z**	23	2100
**ZE**	-	310
**ZZr**	23	1750
**ZZrE**	23	270
**ZW**	23	392
**ZWE**	24	110
**ZV**	27	1820
**ZVE**	45	200
**B**	25	1820
**BE**	-	350
**BZr**	25	1620
**BZrE**	25	250
**BW**	25	340
**BWE**	26	29
**BV**	29	1710
**BVE**	47	114

[a] Ratio Si Metalic cation in the zeolite framework determined by ICP-AES.

[b] μmol NH_3 desorbed from 110 up to 550°C per g of sample

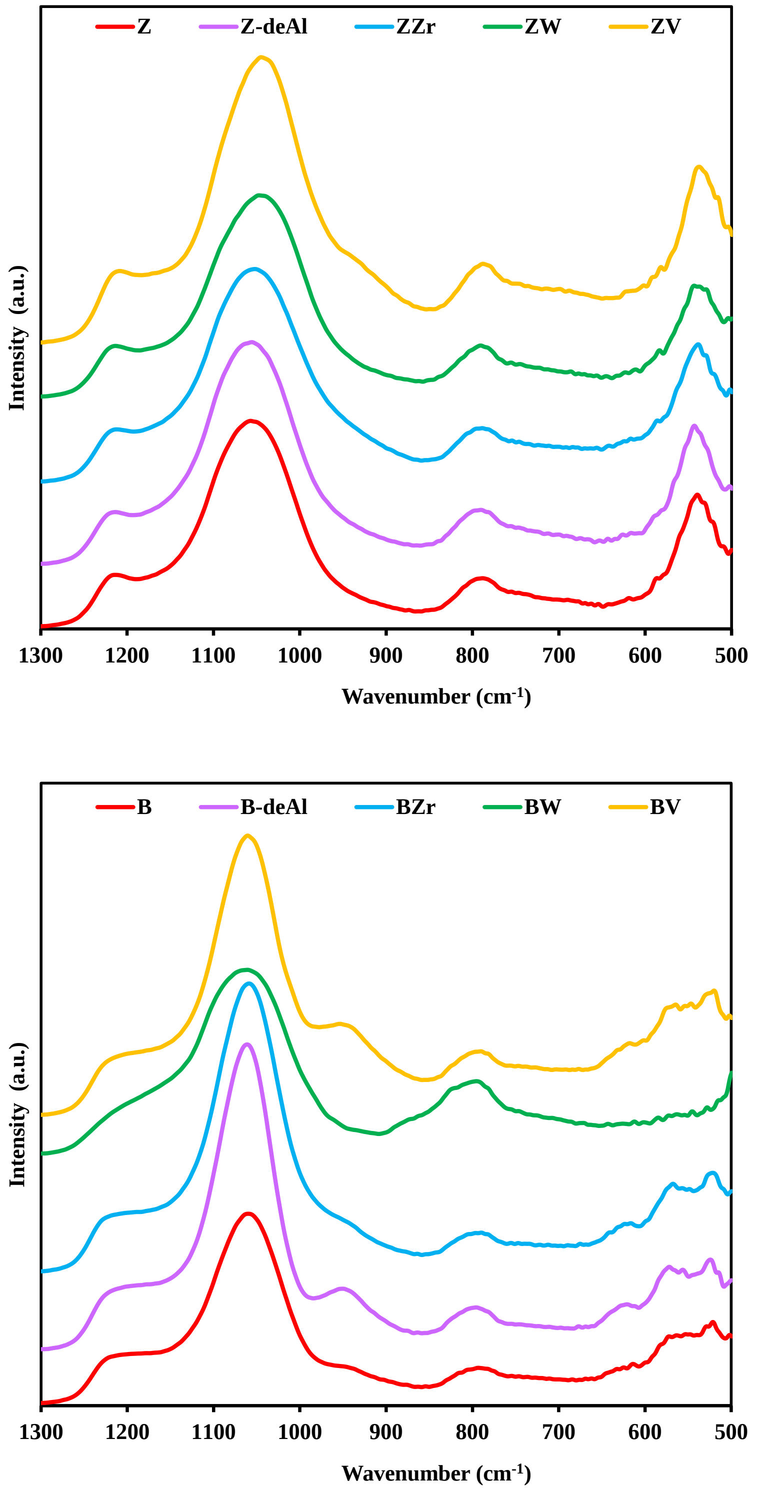

Figure S1. DRIFTS spectrum of Zeolites sample series of ZSM-5 and BETA

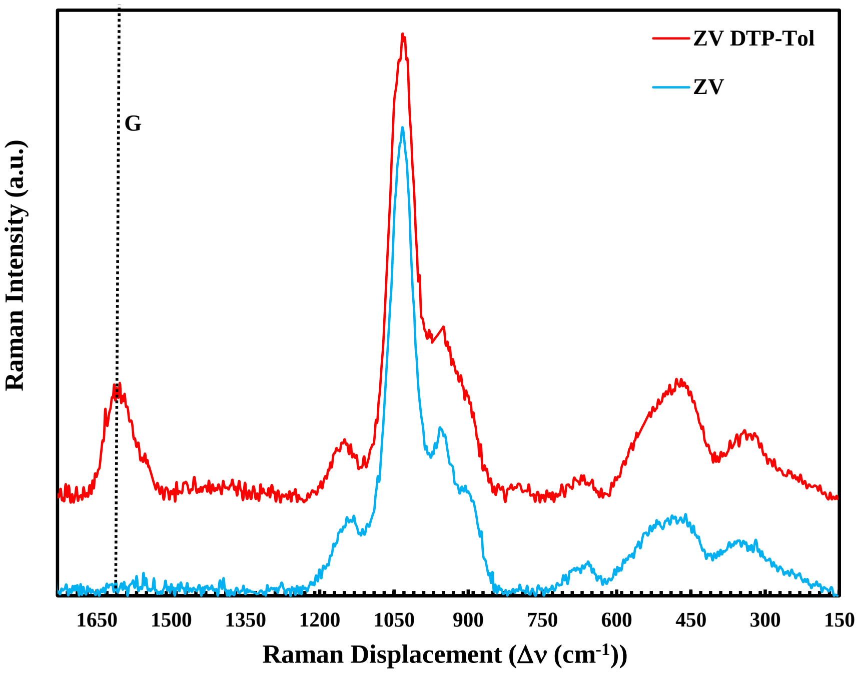

Figure S2. Raman spectra visible at 325 nm of zeolite ZV and ZV before toluene DTP

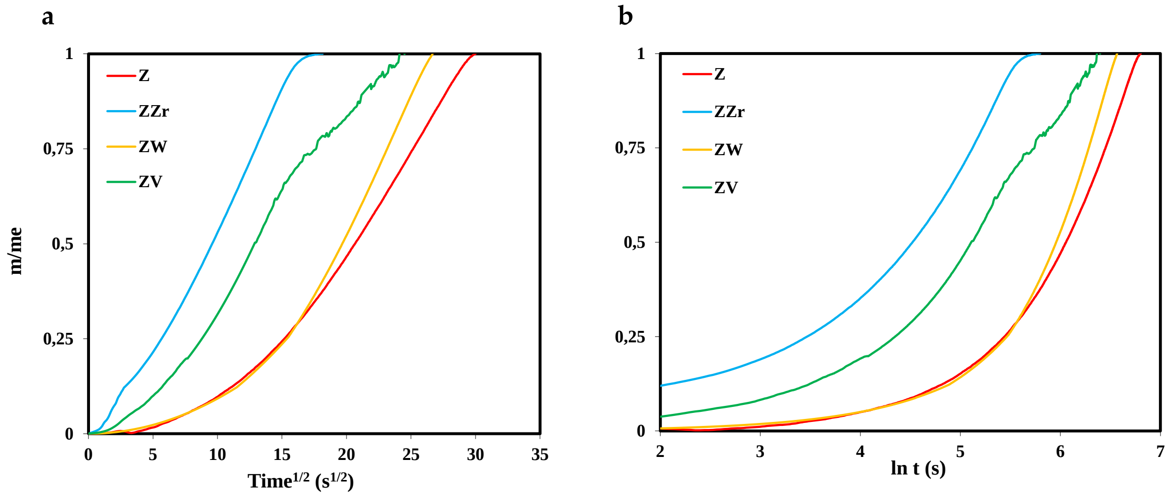

Figures S3. Kinetic curves obtained by application of analytical equation Fick´s Law a) and the Elovich equation b) to toluene adsorption data on ZSM-5 series.

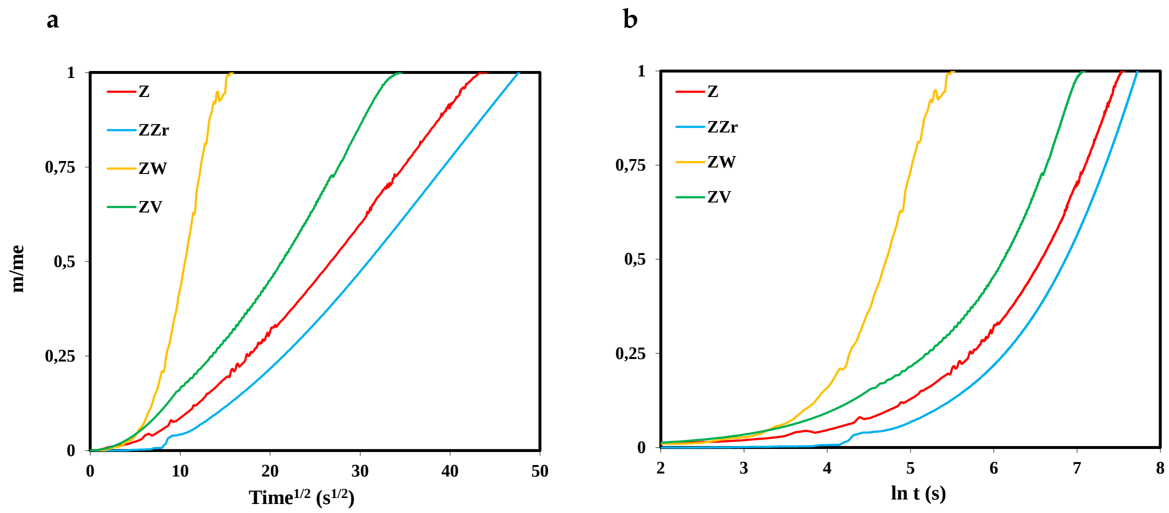

Figures S4. Kinetic curves obtained by application of analytical equation Fick´s Law a) and the Elovich equation b) to propene adsorption data on ZSM-5 series.

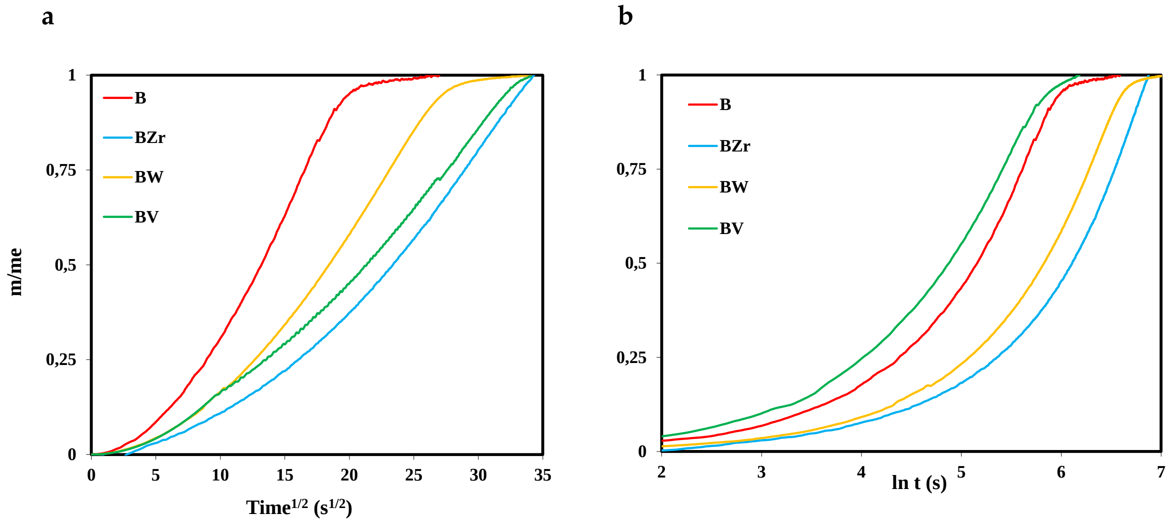

Figures S5. Kinetic curves obtained by application of analytical equation Fick´s Law a) and the Elovich equation b) to toluene adsorption data on BETA series.

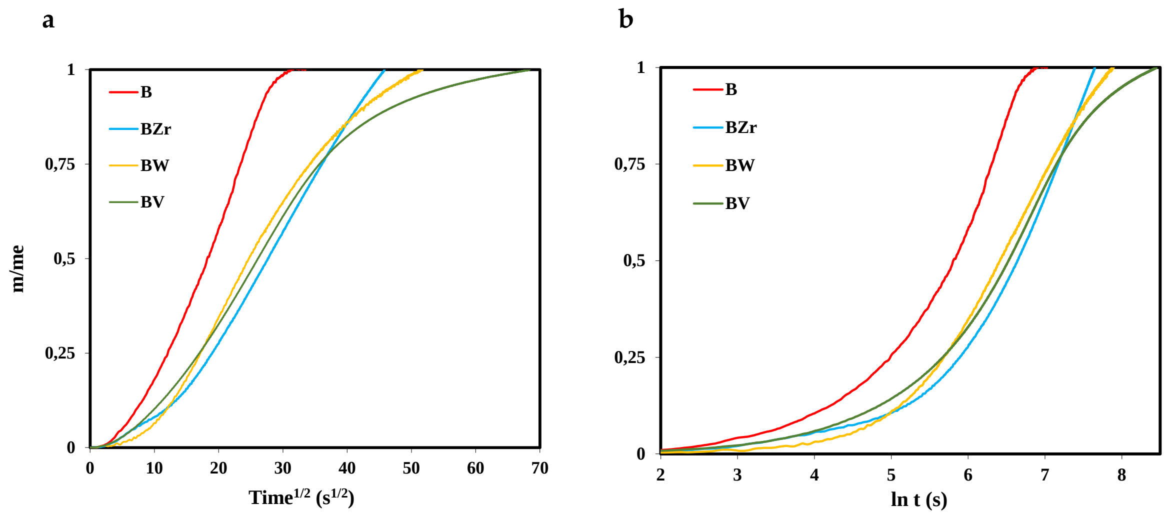

Figures S6. Kinetic curves obtained by application of analytical equation Fick´s Law a) and the Elovich equation b) to propene adsorption data on BETA series.

Two kinetic toluene and propene adsorption models have been applied to the experimental data in Figures 4-5. The resulting curves are plotted in Figures S4-S6. Figures S4-S6 a) contains the profiles obtained assuming that the HCs adsorption rate is controlled by molecular diffusion throughout the microporous network of the zeolites, whose rate is described by the analytical solution for Fick's Law: $m/m_e = k_s\,(t\,D / r^2)^{1/2}$

where m and m_e indicate the quantities adsorbed at time t and at equilibrium, D is the diffusion coefficient, and r is the length path. On the other hand, Figures S4-S6 b) contains the profiles obtained assuming that the HCs adsorption rate is controlled by the chemisorption of vapor on active sites, whose rate can be described by the Equation of Elovich: $m/m_e = a + (1/b)\ln(t+t_0)$

where a and b are temperature-dependent constants.

In general terms, completed linear trends are not observed, indicating that pure adsorption mechanism does not takes place, thus neither the molecular diffusion in the zeolite micropores nor

the chemisorption in the acid sites determine the overall adsorption rate. Nevertheless, linear trends are observed after an early period in Figures S4-S6 a), related to the molecular diffusion mechanism. This is more extends in the case of propene adsorption, whereas with toluene adsorption the range of m/m_e values is lower. This suggests that diffusion of toluene inside of microporosity is also influenced, not only by the potential adsorption of the micropores but also by the interaction with their acid sites presented on the pore walls.
